# Editorial: Abiotic stress signaling in plants: functional genomic intervention, volume II

**DOI:** 10.3389/fpls.2023.1334467

**Published:** 2024-01-16

**Authors:** Ashish Kumar Srivastava, Amita Pandey, Maik Böhmer, Girdhar Kumar Pandey

**Affiliations:** ^1^ Nuclear Agriculture and Biotechnology Division, Bhabha Atomic Research Centre, Mumbai, Maharashtra, India; ^2^ Homi Bhabha National Institute, Mumbai, India; ^3^ Shriram Institute for Industrial Research, Delhi, India; ^4^ Institute of Molecular Biosciences, Faculty of Biological Sciences, Goethe University Frankfurt, Frankfurt, Germany; ^5^ Department of Plant Molecular Biology, University of Delhi South Campus, New Delhi, India

**Keywords:** abiotic stress, stress signaling, stress tolerance, functional genomics, crop productivity

Abiotic stresses such as high temperature, low temperature, drought, and salinity limit crop productivity worldwide. Understanding plant responses to these stresses is essential for the engineering of stress tolerance in plants. In Arabidopsis, the signal transduction pathways have been elucidated for perceiving and responding to different abiotic stress conditions (Zhu, 2016). A significant portion of plant genomes codes for proteins involved in signaling such as receptors, sensors, kinases, phosphatases, transcription factors, and transporters/channels (Pandey, 2020; Chen et al., 2021). Despite decades of physiological and molecular efforts, knowledge about how plants sense temperature fluctuations, drought, water submergence, and salinity signals is still limited. Further, the stress combinations pose another layer of complexity to this problem (Zandalinas et al., 2023). One of the major constraints hampering our understanding of these signal transduction processes has been the slow pace of gene-function studies in plants (Shinozaki and Yamaguchi-Shinozaki, 2022). In this post-genomic era, investigation and understanding of multiple genes and gene families regulating a particular physiological and developmental aspect of plant’s life cycle is of utmost importance (Pandey et al., 2016; Baekelandt et al., 2023; Tyczewska et al., 2023). With a holistic understanding of the signaling pathways involving not only one gene but multiple genes from a signaling cascade, plant biologists can lay a foundation for designing and generating future crops that can withstand environmental stresses without jeopardizing the crop yield.

In the present Research Topic, various novel genes, mainly transcription factors, have been identified and characterized, that can serve as promising candidates for enhancing abiotic stress tolerance in crops. Han et al. have functionally characterized *GmWRKY21* that encodes a nuclear-localized WRKY transcription factor. *GmWRKY21* is expressed across different tissues including roots, leaves, and flowers of soybean and is substantially upregulated in response to aluminum stress. The overexpression of *GmWRKY21* in Arabidopsis increased the root growth of seedlings in transgenic lines under aluminum stress, which coincided with higher proline and lower malondialdehyde (MDA) accumulation and upregulated expression of aluminum stress-specific as well as general stress marker genes. Similarly, Wang et al. identified *GLYCINE MAX ABA REPRESSOR1* (*GmABR1*) encoding an Ethylene Response Factor (ERF) transcription factor as a positive regulator of aluminum stress tolerance in *Arabidopsis thaliana*. The Basic Leucine Zipper (bZIP) family represents another large family of transcription factors that play an important role in mediating plant responses to abiotic stresses. Samtani et al. identified two homeologs of wheat *OCS-ELEMENT BINDING FACTOR1* (*TaOBF1-5B* and *TaOBF1-5D*) as heat-responsive TabZIP members. The heterologous overexpression of *TaOBF1-5B* resulted in enhanced thermotolerance in transgenic *Arabidopsis thaliana* and *Oryza sativa*. TaOBF1-5B has been shown to interact with HEAT SHOCK PROTEIN70 (TaHSP90) in the nucleus and TaSTI (a co-chaperone) in the nucleolus. MYBs (myeloblastosis) are another class of transcription factors that play a pivotal role in regulating anthocyanin synthesis. Liu et al. identified and characterized *PqMYB113* from the leaves of *Paeonia qiui*, a wild species of tree peony native to China. The overexpression of *PqMYB113* resulted in increased anthocyanin accumulation in *Arabidopsis thaliana* and *Nicotiana tabacum*.

Similarly, Jin et al. reported on a homolog of the RelA/SpoT protein (HpRSH) from *Haematococcus pluvialis*, which catalyzes Mg^2+^-dependent synthesis and hydrolysis of guanosine tetraphosphate (ppGpp). The transcription of *HpRSH* was significantly upregulated by environmental stresses, such as darkness, high light, nitrogen limitation, and salinity stress. Apart from these positive regulators, Ge et al. identified *STRESS-INDUCED DUF1644* (*OsSIDP301*), a member of the DUF1644 (Domain of unknown function 1644) family, as a negative regulator of salt stress and grain size in rice. OsSIDP301 determines salt stress tolerance by modulating genes involved in the salt-response and ABA signaling pathways. Moreover, OsSIDP301 interacts with OsBUL1 COMPLEX1 (OsBC1), which positively regulates grain size in rice.

Another approach to identify the stress-responsive gene(s) and associated physiological processes is to compare the contrasting accessions/varieties. Gao et al. performed the phenotypic and metabolic level comparison of two oat cultivars, Baiyan7 (BY, tolerant cultivar) and Yizhangyan4 (YZY, sensitive cultivar) under saline-alkali stress, which is a major detrimental abiotic stress factor for reducing agricultural productivity. Saline-alkali induced metabolites were mainly associated with enhancements in energy metabolism and accumulations of organic acids. The differential abundances of these metabolites revealed that BY showed better adaptability to saline-alkali stress compared with YZY cultivars. Similarly, Dutta et al. performed a genome-wide analysis and identified 10 cytosine-5 DNA methyltransferases (C5-MTases) and 8 demethylases (DeMets) in potatoes. Based on comparative expression profiling in heat-sensitive (HS) and -tolerant (HT) genotypes, several positive [*SELF PRUNING 6A* (*StSP6A*) and *BEL1-LIKE HOMEODOMAIN* (*StBEL5*)] and negative [*SELF PRUNING 5G* (*StSP5G*), *SUCROSE TRANSPORTER4* (*StSUT4*), and *RELATED TO AP2 1* (*StRAP1*)] regulators were identified that regulate potato tuberization under high-temperature stress.

Genome-wide analyses of gene families followed by ranking analysis have also been reported in the Research Topic to identify the key genes. For instance, CALCIUM-DEPENDENT PROTEIN KINASES (CDPKs) are a major group of calcium (Ca^2+^) sensors in plants. CDPKs play a dual function of “Ca^2+^ sensor and responder.” Deepika et al. identified commonly induced *CaCDPKs* in response to drought, salt, and abscisic acid in chickpea. Rajasheker et al. reported the stress-responsive behavior of proline-rich proteins and hybrid proline-rich proteins super family genes in *Sorghum bicolor*. Further, “omics” based approaches have also been used for the identification of genes and regulatory networks associated with nitrogen (N) addition in *Solidago canadensis* (Wu et al.), hydrogen peroxide stress in pepper (Tang et al.) and coleoptile senescence in rice (Sasi et al.).

Apart from genetic engineering, priming with microbes and chemicals are also used as alternatives to enhance plant performance under stress conditions. Li et al. demonstrated the protective role of *Piriformospora indica* (a root endophytic fungus) in wheat against *Rhizoctonia cerealis* and *Fusarium graminearum* infections. RNA-seq suggested that transcriptome changes caused by *F. graminearum* were more severe than those caused by *R. cerealis*. Further, the supplementation of *P. indica* reduced the number of differentially expressed genes (DEGs) by 18% and 58% in wheat injected with *F. graminearum* and *R. cerealis*, respectively, indicating the activation of specific mechanisms. Further, the DEGs related to disease resistance, such as *WRKYs* and *MAPKs* were upregulated in wheat by *P. indica* colonization.

In addition to the research articles, the Research Topic also featured fundamental reviews. For instance, Lim et al. reviewed the core signaling and function of ABA, which is one of the major stress hormones in plants. In addition, the role of non-hormone signal mediators was also highlighted. Li et al. provided a comprehensive insight into sulfur dioxide, which has recently emerged as a signaling mediator in plants. Finally, Khan et al. reviewed the recent advances in the application of thermal techniques for inducing stress-priming during the seedling stage, which is another non-genetic approach.

Taken together, the Research Topic highlights the novel research findings in the field of abiotic stress signaling in plants, that may pave the path for developing “stress-tolerant” crops in the near future.

## Author contributions

AS: Writing – original draft, Writing – review & editing. AP: Writing – review & editing. MB: Writing – review & editing. GP: Conceptualization, Funding acquisition, Writing – original draft, Writing – review & editing.
